# The Presentation to Sir E. Cooper Perry

**Published:** 1920-02-14

**Authors:** 


### THE PRESENTATION TO SIR E. COOPER PERRY.

### Detailed Report of the Speeches.*

**Dr. Lauriston Shaw,** introduced as Sir Cooper's oldest friend and colleague, spoke as follows :—

SIR COOPER PERRY,—Forgive me if I first thank this assembly of your fellow-workers at Guy's for entrusting to me an agreeable—if somewhat difficult—task. For I have been asked to act as their spokesman this afternoon, and now I must tell you on whose behalf I am speaking. I am speaking on behalf of that body of persons whom the Editor of the *Gazette*, in a " Passim " note this week, with his usual happy choice of language, calls the Hospital Community. It is a community bound together by a common privilege of addressing you or at least of speaking of you, familiarly and affectionately, as the " Super " ; a community bound together to-day by a common grief that they are so soon officially to lose this privilege, though I venture to think that many of us will long continue to exercise it, whatever new titles may be more appropriate to your future position or to any well-deserved honours that may be hereafter conferred upon you.

Every section of this community, medical, surgical, dental and specialist staff, nursing staff, staff of the medical and dental schools, administrative staff, past and present students in the schools, and last, but not least, the Works Department, vies with each other in a desire to express its regret that you are so shortly to vacate your office of Superintendent of the hospital, its pleasure that you have been made a Governor, and its earnest hope for your happiness in your new work and ultimately in your retirement, an event which, having regard to your abounding energy and love of work, seems indeed very remote.

Representatives from each section are present here to-day, and if some sections are less numerously represented than others I am sure it is to be attributed not to any want of enthusiasm for the object of this meeting, but to a certain shyness in which both you and I can sympathise with them. We are indeed grateful to you for allowing us this opportunity for saying our farewells and good wishes to you in person, knowing as we do how averse you are from all functions and ceremonies in which you are called upon to take a prominent part.

In the letter in which I was told that the committee arranging these proceedings had expressed a wish that I should act as spokesman, it was said that I had been chosen because it was believed that I was your oldest friend at Guy's and had been more closely connected than anyone else with your work here. I do not know how this may be, but I am sure we are very old friends and have done much pleasant work here together. Perhaps I may remind you of the beginning of our friendship. Thirty-three years ago you and I, unknown to each other, were applicants for the much-coveted post of assistant physician. The Governors of the hospital in their wisdom declined my application and accepted yours. A few mornings later when going round the wards with my class as medical registrar, bemoaning, I daresay, the hard fate which had ordained that the unusual, if not unprecedented, action of the Governors in selecting for the staff one who had not received his medical education at Guy's, should have apparently cut short my ambitions, Mr. Lushington, the then Treasurer, came into the ward and calling me from the bedside, introduced me to the new assistant physician. From that day to this I have never for a moment ceased to be profoundly grateful to the Governors for postponing my election to the staff, for by this action they secured for me one of the longest, closest, and happiest friendships of my life, and secured for my beloved hospital what, I believe, its future historians will recognise as the greatest piece of good fortune that has befallen it during the first two centuries of its existence. Ever since that date you have devoted to the service of the hospital your high intellectual attainments, your talents as a teacher, but above all, your incomparable powers of construction, organisation, and administration. During all this time you have lived amongst the Hospital Community, and for a large part of the time you have been accompanied by Lady Perry and your daughter, who have, like you, endeared themselves to many of its members, and to whom also we desire to convey our expressions of regret at losing their society and of good wishes for their future happiness.

There was a tale current when I was first a student here, which some of you have, no doubt heard. A senior member of the staff was taking round an American who did not appear to be very appreciative of what was being shown to him. Coming to the steps of the colonnade and looking out into the front quadrangle, as a last effort, our former colleague said, " And here you see the statue of our founder, Thomas Guy, standing amidst the buildings exactly as they were when he planned them 150 years ago and unaltered since the first patient was admitted just a week after his lamented death in the year 1725." To which the American replied, " I guess if your old man Guy had built his hospital in our country as long ago as that we would have pulled the whole darned thing down two or three times by now and rebuilt it—better each time." Since you have been Superintendent, Guy's has been practically rebuilt, but we are grateful to you that you have not " pulled the whole darned thing down." So far as the progress of the standard of comfort of the patients and the demands of advancing science have allowed, you have retained for us the original structure and its best architectural features, so that we may ever be reminded of our ancient traditions and of our glorious past. The additions put up under your guidance and inspiration are numerous and now quite indispensable. The residential college, the dental school, the new school buildings, the laundry and central power and light station, the nurses' hostel, the pathological institute, and many another new or enlarged department, all owe their origin and completion to your inspiration and energy. Only yesterday some of us attended the formal opening of the buildings of the Salomons Infant Welfare Centre, destined, let us hope, to be the pioneer of many further efforts here in the direction of preventive medicine. You are leaving us a yawning chasm in the park, the foundations for additions to the nurses' quarters and for a new building for the electrical and remedial exercise department. You have already planned for us at the top of Hunt's House two new floors to accommodate a baby's ward, a new clinical extension of the midwifery ward, and wards for other special departments.

There are some who think the best and most permanent memorial of your work here as Superintendent will be these buildings. Others, with heads for figures and high

* By kind permission of the Editor of *Guy's Hospital Gazette*.

**Presentation to Sir E. Cooper Perry—(*continued*).**

finance, think you may best be remembered by our banking account during the last thirty years. They point to the hundreds of thousands raised and spent on these permanent works and to the half million that has been added to the Re-endowment Fund.

But things of the spirit are more enduring than things of the flesh, and human societies often outlive the bricks and mortar in which they are housed or their bank balances, however large and cautiously invested. Perhaps a still more lasting memorial of you than buildings or money may be the atmosphere which you have so largely helped to create for this great hospital community to work in. I am old enough to remember the time when our community was sadly rent by discord and dissensions. I am confident that there is no busy body of workers of equal size to-day whose mutual relations are so full of peace and good fellowship. You would be the first to recognise how largely this happy condition is due to those in still higher authority over us. But by example and precept you have taught us to make the interest of the individual subservient to the interest of the institution, and that the welfare of each department must be subordinated to the welfare of the hospital as a whole. You are binding us still closer to-day by inspiring us with a desire to render to you a common homage on your retirement. . . .

**Sir Cooper Perry** replied :

Dr. Fawcett, Ladies and Gentlemen, Friends all!—I have to thank you on behalf not only of myself, but of Lady Perry and my daughter, for the very handsome presents you have made me, and still more for the kindly spirit in which you are speeding the parting Superintendent.

Kindness, indeed, I have invariably experienced at Guy's from the first day I entered it some thirty-two years ago, when, a stranger from another and at that time somewhat despised hospital, I met Sir Thomas Stevenson on the top of the steps of the Colonnade, and, as is the fashion of Guy's, regarding me critically, but not unkindly, he said, "Now we have you, we shall expect you to work." I humbly, and I hope truthfully, replied, "That I can do."

No sooner was I here than I realised that Guy's was a place where people worked. I saw the proofs of the quiet labours of generations in the Post-mortem Records, in the great collection in our Pathological Museum, and in the accumulated piles of clinical reports; and Mr. Wells used in his later years to repeat the remark which I made when I had been here some months and was meditating various improvements, that I believed the old place had a sound constitution. And I based my belief on the evidence all around me of steady, honest work, which was the joy of those before me and of my contemporaries, a spirit alive as ever in the present generation.

Now, work may be done for many motives—for the honest motive of obtaining a competence, for the less worthy motive of advertisement; but the work we see and admire here is work done with no thought of advertisement about it, done by a worker with an ideal, an ideal which Bacon expresses in the words "the glory of the Creator and the relief of man's estate." And the labours of how many of us go to the task which the modern hospital sets out to fulfil!

I often think of Guy's as a coral reef, gradually raised above the water by the infinite minute accretions of toil contributed by every one of us. How many are the labourers all working to one end! You see us based upon the Works Department, the servant, perhaps, at times, the master of us all; the laundry, where we are washed more or less perfectly; Tabard House, where not with entire success they endeavour to carry out the Apostle's instruction that women should study to be quiet, but where they work as girls love to work when their heart is in it; the kitchens, that cheap mark of satire; the bakehouse; the counting house; the Steward's department and stores; the Superintendent's office; the Matron's office; the sewing-room, uncompromising in its exposure of any failure by a nurse to conform to the only types there recognised as normal in the human female. All these departments I enumerate, and the list is not exhausted, before we even approach our medical school and students, the sisters and nurses, the patients and the wards, with their hierarchies, male and female, from the Physician to the Household, or the Surgeon to the King, to the newest probationer, who, if she only knows it, is entering on the happiest three years of her life. Such is the complexity of the machine, so great the task, to carry on the commonwealth.

Now, this work of ours, done for an ideal directed to the relief of man's estate, draws us together as a family. We work together, we play together, at least some of us play. We share each other's sorrows and, what is often harder, each other's successes. If we win football matches —and we did sometimes win them even before the South Africans came over—we rejoice, from the Senior Physician to the Lab. boy, and this community in work and play and in ideals makes us stick together in a fashion which is proverbial all the world over. Guy's men and Guy's women are credited with a clannishness binding us as closely as if we all came from the other side of the Tweed. Long may these ties of work and common aims remain firm fixed in the Borough!

One fault, however, I have to find with those who have said such kind things about me; that, as is perhaps inevitable, they have exaggerated the importance of myself, who am just one single cog in the great machine. Not one of the achievements they lay to my charge could have been accomplished except as part of that machine. For example, the College, which was projected by Dr. Addison, actually came into being because we all pulled together—Mr. Lushington, our Governors, and staff—and took the risks. The Dental School owes more to Mr. Newland-Pedley than to myself, and what a small affair it was when we left it compared with the magnificent structure into which it has developed through those who came after us. Our great appeal in 1896 : what would have come of it without Mr. Bonsor, Sir Alfred Fripp, and Mr. Wells?

None knows better than myself how little I have done, nor should I be content to be thus praised did I not make it freely known that the praise you gave me is due in a far greater degree to the company of faithful workers and loyal sons and daughters of Guy's whom it was my lot to have as my companions and helpers through the whole of my service.

I am fortunate that the end of my time here has brought me what it ought to be the fervent prayer of all of us to find at the close of the long struggle with ourselves and with circumstances—a disposition to happiness, a composed spirit to which age has made things plain, an unrebellious temper, and hopes undimmed for mankind. These things I count myself lucky in possessing, for I am confident that the work to which I am going at the London University is the work to which Providence has called me, that in leaving Guy's I am doing what is best for Guy's, and that I am confiding the task which was mine to young and willing hands who will be proud of the past of Guy's, who will endeavour to show themselves worthy of their great inheritance. Once more I thank you.

